# Oligodendrogliomas tend to infiltrate the frontal aslant tract, whereas astrocytomas tend to displace it

**DOI:** 10.1007/s00234-023-03153-6

**Published:** 2023-05-02

**Authors:** M. J. F. Landers, H. B. Brouwers, G. J. Kortman, I. Boukrab, W. De Baene, G. J. M. Rutten

**Affiliations:** 1 Department of Neurosurgery, Elisabeth-Tweesteden Hospital Tilburg, Tilburg, The Netherlands; 2 Department of Cognitive Neuropsychology, Tilburg University, Tilburg, The Netherlands; 3 Department of Neuroradiology, Elisabeth-Tweesteden Hospital Tilburg, Tilburg, The Netherlands

**Keywords:** MR-tractography, Low-grade gliomas, Oligodendroglioma, Astrocytoma, Subcortical tract, Frontal aslant tract

## Abstract

**Introduction:**

MR-tractography is increasingly used in neurosurgical practice to evaluate the anatomical relationships between glioma and nearby subcortical tracts. In some patients, the subcortical tracts seem displaced by the glioma, while in other patients, the subcortical tracts seem infiltrated without displacement. At this point, it is unknown whether these different patterns are related to tumor type. The aim of this exploratory study was to investigate whether tumor type is related to the spatial tractography pattern of the frontal aslant tract (FAT) in low-grade gliomas (LGGs).

**Methods:**

In 64 IDH-mutated LGG patients, the FAT was generated using a pipeline for automatic tractography. In 41 patients, the glioma adjoined the FAT, and four blinded reviewers independently assessed the following two dichotomous categories (yes/no): (i) glioma displaces the tract, and (ii) glioma infiltrates the tract.

**Results:**

Fisher’s exact tests demonstrated strong and significant positive associations between displacement and astrocytomas (*p* = .002, φ = .497) and infiltration and oligodendrogliomas (*p* = .004, φ = .484). The interobserver agreement was good for both categories: (i) κ = 0.76 and (ii) κ = 0.71.

**Conclusion:**

High sensitivity but low specificity for displacement in astrocytomas demonstrates that in the case of an astrocytoma, the tract is most likely displaced, but that displacement in itself is not necessarily predictive for astrocytomas, as oligodendrogliomas may both infiltrate and displace a tract. Overall, these results demonstrate that oligodendrogliomas tend to infiltrate the nearby subcortical tract, whereas astrocytomas only tend to displace it.

**Supplementary Information:**

The online version contains supplementary material available at 10.1007/s00234-023-03153-6.

## Introduction

MR-tractography is increasingly used as a clinical tool to visualize subcortical tracts and to plan brain tumor surgery [1]. The anatomical relationship between a brain tumor and nearby subcortical tracts can be used to determine a safe surgical corridor and plan and is predictive of the extent of resection (EOR) [2, 3]. This is especially relevant in the case of low-grade glioma (LGG), as LGGs have a diffuse growth pattern, and the EOR is strongly related to survival time [4]. Determining the EOR of LGGs therefore requires a careful balance between the removal of tumor tissue and sparing functional subcortical brain tissue. Therefore, when determining the EOR, spatial information on the anatomical relationship may provide valuable insight. Currently, a clear and uniform clinical classification system to classify the spatial patterns of subcortical tract alterations specifically for LGGs is lacking. For all types of brain tumors (low- and high-grade gliomas, metastases), three non-uniform systems have been reported [5–7]. These systems distinguish, amongst others, between the patterns of displacement and infiltration but also rely their classification on fractional anisotropy (FA), mean diffusivity (MD), and/or directional color maps. From clinical experience, it caught our attention that the different spatial patterns may also be linked to the 1p19q status of the LGG, whereby in case of displacement, the histopathological examination resulted more often in the absence of a 1p19q codeletion (astrocytoma), while in case of infiltration, this resulted more often in the presence of a 1p19q codeletion (oligodendrogliomas). In the field of radiomics, several attempts have been made to classify 1p19q status in LGGs. A recent systematic review that investigated MRI radiomics and 1p19q deletion demonstrated that texture-based radiomics could classify 1p19q status in IDH-mutated LGGs with a maximum sensitivity of 85% and specificity of 77%, but that clinical application was limited due to the high heterogeneity between each of the radiomic pipelines [8]. In addition, none of the papers included MR-tractography for classification of the 1p19q status. Currently, it remains unknown whether the different clinically observed spatial patterns are related to the 1p19q status of LGGs. The aim of this exploratory study was to investigate this hypothesis for the frontal aslant tract (FAT) in LGG patients, whereby we hypothesize that displacement is associated with astrocytomas and infiltration with oligodendrogliomas.

## Methods

**Fig. 1:**
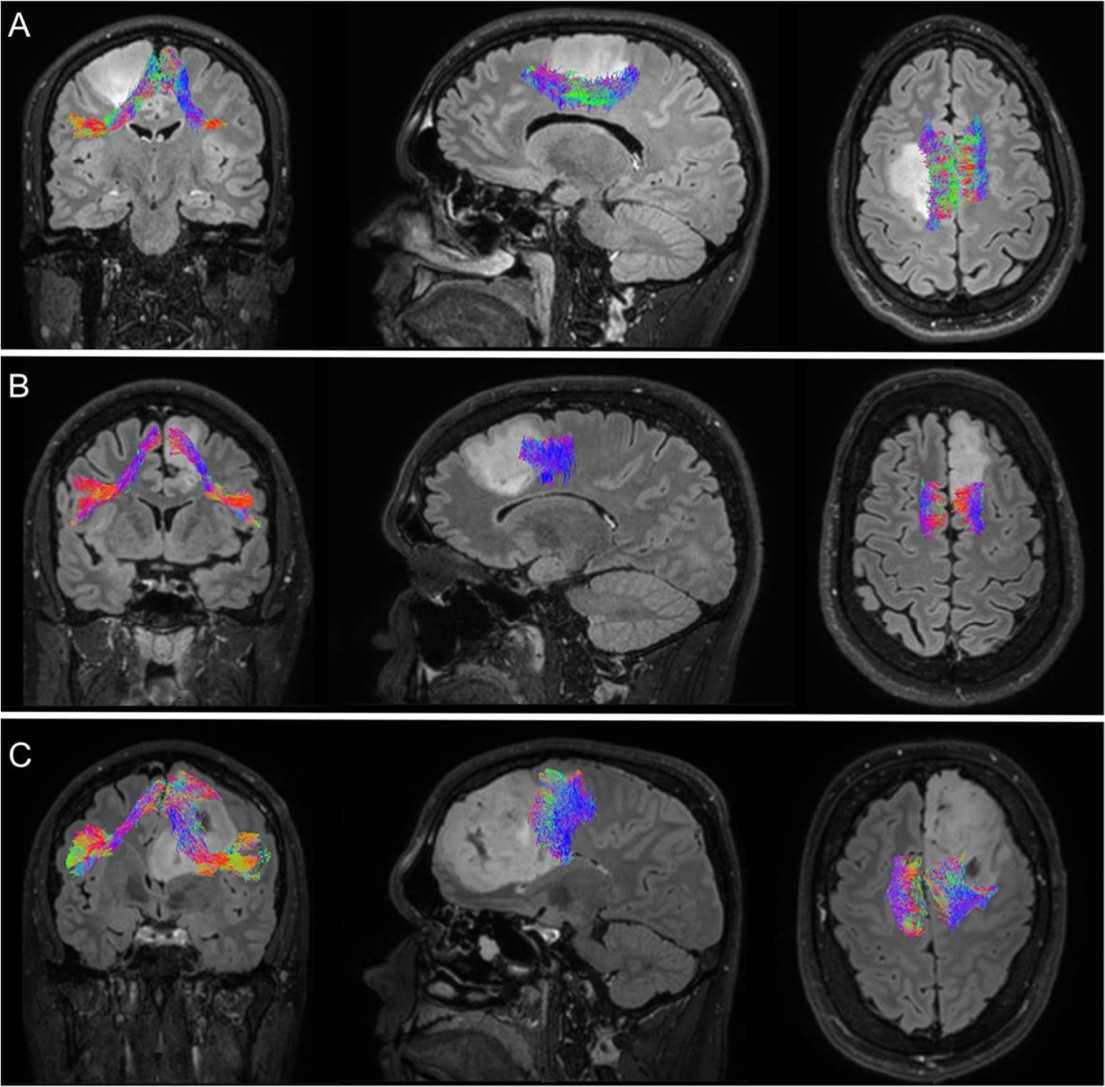
**A–C** Coronal, sagittal, and axial view of three different subjects as examples. **A** Example in which the frontal aslant tract was displaced but was not infiltrated. **B** Example in which the FAT was infiltrated but not displaced. **C** Example in which the FAT was displaced and infiltrated

We retrospectively analyzed patients with frontal IDH-mutated low-grade gliomas, from whom DW-MRI was acquired in the week before resection in the period between April 2011 and July 2021. Patients were included if histopathological examination confirmed an oligodendroglioma (1p19q codeletion present) or astrocytoma (absence of 1p19q codeletion). DWI scans were acquired using a Philips Achieva 3T MRI-scanner (*b* = 1500, 50 diffusion weighting directions, 6 *b* = 0 images, 2 mm isotropic voxel size). Probabilistic tractography of the FAT was performed using a pipeline for subject-specific automatic reconstruction of white-matter pathways (for the tractography protocol, see Appendix). This automated pipeline uses the MRtrix software package for the constrained spherical deconvolution-based iFOD2 method with *tckgen* [9]. The shortest distance from the glioma to the FAT was computed, and patients in which the glioma was located beyond 1 cm of the FAT were excluded. Four blinded examiners (ML, HB, GK, IB) independently assessed the following two dichotomous categories (yes/no): (i) glioma displaces the tract; (ii) the glioma infiltrates the tract. The FAT was classified as displaced in case the tumor affected the course of the FAT and ran asymmetrically as compared to the contralateral hemisphere. The FAT was classified as infiltrated in case the tract ran partly through the tumor (see Fig. 1A–C for examples of the two categories). Differences in assessments were adjudicated by consensus.

Descriptive statistics were performed for the following patient characteristics: age, sex, affected hemisphere, tumor volume, and FA and MD of the tumor. We statistically compared the characteristics with independent samples *t*-tests or Mann-Whitney *U* tests (continuous variables, depending on data distribution) and Chi-square tests (categorical variables). The significance level was set at 0.05. Interobserver agreement was calculated between the four examiners per category using Fleiss’ kappa. Fisher’s exact tests were used to investigate whether there were significant associations between the two categories and histopathological tumor type. In the case of significant associations, we calculated sensitivity, specificity, positive and negative predictive value, and accuracy.

## Results

In 41 out of 64 patients with a frontal LGG, the glioma was located within 1 cm of the FAT. Comparison of the patient characteristics (Table 1) between oligodendrogliomas and astrocytomas demonstrated a significant difference in age and MD, with a lower mean age in the astrocytoma group (*p* < .05*)* and a lower mean MD in the oligodendroglioma group (*p* < .05). The groups did not differ significantly on any of the other patient characteristics. In six cases, differences in assessment of the spatial categories were adjudicated by consensus. In 31 cases, there was displacement, and in 24 cases, the FAT was infiltrated. There were no cases in which there was no infiltration or displacement. Fisher’s exact tests demonstrated strong significant positive associations between displacement and astrocytomas (*p* = .002, φ = .497) and infiltration and oligodendrogliomas (*p* = .004, φ = .484). The interobserver agreement was good for both categories, (i) κ = 0.76 and (ii) κ = 0.71. For the significant associations, sensitivity, specificity, positive and negative predictive value, and accuracy are presented in Table 2. For raw data see supplementary material.

**Table 1:** Patient characteristics (*N* = 41)

		Oligodendroglioma (*N* = 19)	Astrocytoma (*N* = 22)
Age mean (SD; range) in years		46.9 (11.5; 29 - 66)*	36.3 (11.3; 21–61)*
Sex (*n*)	Male Female	11 58% 8 42%	14 64% 8 36%
Affected hemisphere (*N*)	Right Left	6 32% 13 68%	10 46% 12 55%
Tumor volume median (Q1;Q3)^1^ in cm3		35.0 (14.4; 61.0)	28.6 (10.4; 70.1)
Tumor FA mean (SD; range)		.149 (.028; .088–.197)	.143 (.026; .097–.196)
Tumor MD^1×10e−3^mean (SD; range)		.128 (.013; .109–.163)*	.142 (.019; .113–.181)*

^1^Quartile 1, median of the lower half of the dataset; quartile 3, median of the upper half of the dataset

^*^p < .05

**Table 2:** Spatial pattern for the prediction of tumor type

Total *N* = 42	Displacement and astrocytoma *N* = 21	Infiltration and oligodendroglioma *N* = 16
Sensitivity	95.5%	84.2%
Specificity	47.4%	63.6%
PPV	67.7%	66.7%
NPV	90.0%	82.4%
Accuracy	73.2%	73.2%

*N*, number of patients; *%*, percentage; *PPV*, = positive predictive value; *NPV*, negative predictive value

## Conclusions

Our results demonstrate that 1p19q status in LGG differently affects the course of the frontal aslant tract in and around the tumor. We found a high negative predictive value (90%) for astrocytomas and displacement, indicating that in the case of infiltration without displacement, it is 90% likely that the LGG is an oligodendroglioma. Vice versa, in the case of displacement without infiltration, it is 82% likely that the LGG is an astrocytoma. The high sensitivity but low specificity for displacement in astrocytomas demonstrates that in the case of an astrocytoma, the tract is most likely displaced, but that displacement in itself is not necessarily predictive for astrocytomas (specificity < 50%). The results demonstrate that this can be explained by the tendency of oligodendrogliomas to both infiltrate and displace a tract. Notably, in case of no displacement, this automatically means that there was infiltration because there were no cases in which there was no displacement and no infiltration.

We can not rule out that the differences in tractography results of the FAT are to a certain extent artifactual and caused by different DWI effects of each tumor type. Future research should replicate the results and investigate whether the different spatial patterns also occur in other subcortical tracts. If our findings are indeed replicable and generalizable, they can have profound implications for pre-operative counselling, choice of surgery type (awake vs. asleep), and perioperative decision-making (e.g., determining EOR) [10]. Future studies should also analyze whether these different patterns are related to postsurgical outcome. Hypothetically, if infiltrated tracts are still functional, this would imply that after complete resection, patients with an oligodendroglioma have more functional deterioration than patients with an astrocytoma, because tracts running through the tumor are more likely to be damaged than tracts that are displaced by the tumor. Therefore, we stress the need for an accurate clinical classification system of tractography patterns to enable further research on the diagnostic and prognostic value of MR-tractography, as it seems that the 1p19q status of LGGs differently influences the spatial pattern of a nearby subcortical tract.

### Supplementary Information

Supplementary file 1 (DOCX 16.1 KB)

## Appendix. Tractography protocol

DWI-MRI data were pre-processed using the MRTrix script *dwifslpreproc*. Probabilistic tractography was performed using the constrained spherical deconvolution-based iFOD2 method with *tckgen*. To seed and restrict tractography of the FAT, estimated regions of interest (ROIs) were identified using SLANT, a method that uses deep learning to generate patient-specific segmentations of 133 anatomical regions [11]. As seed regions, the supplementary motor area (SMA) and pre-supplementary motor area were used. As there are no anatomical landmarks that define the pre SMA, a vertical virtual plane passing through the genu of the corpus callosum was used to determine the anterior boundary of the pre SMA. The target regions used were the pars opercularis and pars triangularis of the inferior frontal gyrus. After tracking, spurious streamlines were filtered out of the tracts using fiber-to-bundle coherence as implemented in Dipy [12].

## Abbreviations

FAT: Frontal aslant tract
DW-MRI: Diffusion-weighted magnetic resonance imaging
LGG: Low-grade glioma
DES: Direct electrical stimulation
EOR: Extent of resection
