# Supplementary material for: Oligodendrogliomas tend to infiltrate the frontal aslant tract, whereas astrocytomas tend to displace it
Source: Neuroradiology. 2023 May 2;65(7):1127–31. doi: 10.1007/s00234-023-03153-6 (PMC10271893; doi:10.1007/s00234-023-03153-6)
Supplement: Supplementary file 1 — (DOCX 16.1 KB) [file 234_2023_3153_MOESM1_ESM.docx]

Supplementary Material

**Supplementary table 1.** Raw scores displacement and tumortype (*N* = 41).

|  | Oligodendroglioma | Astrocytoma | Total |  |
| --- | --- | --- | --- | --- |
| Displacement |  |  |  |  |
| Yes | 10 | 21 | 31 |  |
| No | 9 | 1 | 10 |  |
|  |  | | | |
| Total | 19 | 22 | 41 |  |

**Supplementary table 2.** Raw scores infiltration and tumortype (*N* = 41).

|  | Oligodendroglioma | Astrocytoma | Total |  |
| --- | --- | --- | --- | --- |
| Infiltration |  |  |  |  |
| Yes | 16 | 8 | 24 |  |
| No | 3 | 14 | 17 |  |
|  |  | | | |
| Total | 19 | 22 | 41 |  |

**Supplementary table 3.** Raw scores for displacement and infiltration jointly and tumortype (*N* = 41).

|  | Oligodendroglioma | Astrocytoma | Total |
| --- | --- | --- | --- |
| Displaced and infiltrated | 7 | 7 | 14 |
| Dispaced and  not infiltrated | 3 | 14 | 17 |
| Not displaced and infiltrated | 9 | 1 | 11 |
| Not displaced and  not infiltrated | 0 | 0 | 0 |
|  |  |  |  |
| Total | 19 | 22 | 41 |
